# The impact of restricting price promotions on foods high in fat, sugar and salt on purchasing behaviours: a randomised controlled trial in a simulated online supermarket in the UK

**DOI:** 10.1017/S1368980026103073

**Published:** 2026-07-09

**Authors:** Ru Jia, Filippo Bianchi, Madison Luick, Darren Hilliard, Frances Bain, Lauren Bandy, Ailidh Finlayson, Benji Horwell, Elena Meyer zu Brickwedde, Bobby Stuijfzand, Giulia Tagliaferri, Jordan Whitwell-Mak, Rachel Pechey, Hugo Harper

**Affiliations:** 1 Nuffield Department of Primary Care Health Sciences, University of Oxford, UK; 2 Behavioural Insights Team, UK; 3 NESTA, UK; 4 Nuffield Department of Population Health, University of Oxford, UK

**Keywords:** HFSS, Price, Promotion, Communication, Randomised controlled trial

## Abstract

**Objective:**

Price promotions influence food choices and are disproportionately applied to foods high in fat, sugar and salt (HFSS), consumption of which is a known contributor to poor diet and diet-related non-communicable diseases. This study evaluates the effectiveness of (1) restricting price promotions of HFSS products and (2) allowing such promotions but restricting communications regarding them in changing purchasing behaviours.

**Design:**

Between-subjects randomised controlled trial with three arms: (1) reduced price, labelled a price promotion (‘Usual Practice’); (2) reduced price, not labelled a price promotion (‘No Communication’) and (3) standard price (‘No Promotion’). Participants selected food and drinks for 2 d in a one-off shop, without restrictions on the number of items to purchase or the budget, at a simulated online supermarket. We measured total energy (kcal) in basket, total cost, total number of items, energy density (kcal/100 g) and proportion of the basket that was HFSS.

**Setting:**

Simulated online supermarket.

**Participants:**

Adults representative of the UK population (*n* 9004).

**Results:**

There were no significant differences in energy in baskets between groups, after controlling for age, sex, household income, ethnicity and deprivation. No significant differences were found between groups’ baskets for total cost, energy density or proportion HFSS. Participants in the ‘Usual Practice’ group selected significantly more items than the ‘No Promotion’ group (11·4 *v*. 11·1; *P* = 0·003).

**Conclusions:**

No significant effects of restricting price promotions, or communications of them, were found for purchasing behaviours in a simulated online supermarket. Further price-based interventions, especially in real-world settings, are needed to provide robust policy recommendations.

Consumption of food that is high in fat, salt and/or sugar (HFSS) is a known contributor to poor diet, obesity and associated non-communicable diseases^(1–3)^. To target these items, the UK government introduced legislation restricting the promotion, placement and advertisement of HFSS items^(4)^. Since 2022, HFSS items cannot be placed in prominent locations in stores, including within two metres of checkouts or queuing areas, at the end of aisles, at the store entrance or equivalent places online. Other restrictions on HFSS items, such as restrictions of volume price promotions (e.g. multi-buy offers), came into force in 2025 and a ban on TV advertising before 21.00 or paid-for online advertising in 2026.

Price promotions, i.e. making temporary changes to the price of foods and beverages, are used to market food to consumers, to stimulate sales and consumption^(5)^. Among the different types of price promotions, temporary price reductions (TPR: short-term price reductions such as ‘50p off’ or ‘25 % lower price’) are the most common price promotion in the UK^(6,7)^, with foods and beverages high in sugar more likely to get promoted and receive higher discounts^(8)^. A Public Health England analysis on the role of price promotions in household purchases of food and drinks reported that 18 % of take-home food and drink purchases can be considered incremental as a result of price promotions, with TPR contributing to 24 % of overall spend on take home food and drink in the UK in 2015^(9)^. Similarly, an analysis of retail purchase and price promotions in Scotland reported that in 2016 TPR accounted for 26 % of total calories purchased for take-home food and beverages, and that unhealthy food and drinks were more likely to be purchased while on promotion^(10)^. However, such promotions on HFSS products were not within the scope of the regulations on promotions in 2025, despite the evidence that price promotion, especially TPR, significantly increases sales of HFSS foods due to both its widespread application and impact on purchasing behaviours^(8,9,11)^.

It is unclear whether TPR restrictions would be an effective public health policy. Although there is robust evidence that price promotions on unhealthy foods can increase sales, which could then lead to consumption of such foods, there is no direct evidence addressing whether restricting price promotions on unhealthy foods could reduce consumption^(12)^. However, a later study in a simulated online supermarket explored the effects of restricting TPR on HFSS foods completely on purchasing behaviours, reporting 10 % less total energy selected in shopping baskets following the removal of TPR^(13)^. This suggests that restricting TPR on HFSS foods has the potential to influence consumer behaviours, albeit with further studies needed to investigate the robustness of this effect. However, concerns have also been raised regarding whether restricting TPR could impact on the cost of living. There is evidence that price promotions can deliver significant savings for households^(8)^, thus a policy restricting TPR might have implications for food affordability, potentially generating public and/or political backlash. Therefore, it is also important to explore alternative options that might be similarly effective, yet more acceptable. One option might be to allow price promotions on HFSS foods, but restrict the communication of these promotions to consumers (e.g. restricting the signage that alerts the consumers to the reduced price). To our knowledge, there is currently no evidence of the impact of exclusively restricting communications regarding price promotions, without restricting the price promotion itself, on purchasing behaviours. Thus, in this study, we investigated the potential impact of restricting communication of TPR on HFSS items, on food purchasing behaviour. We also investigated the impact of restricting TPR entirely. Specifically, this study set out to investigate:

1. The impact of restricting TPR, or the communication of TPR, on HFSS items, on the total energy (kcal) purchased in an online shopping task;
2. The impact of these interventions on total energy purchased among subgroups (i.e. by income, BMI, and food shopping responsibilities);
3. The impact of these interventions on the total cost of baskets in this shopping task;
4. The public support for such interventions.

## Methods

We reported this study in accordance with the CONSORT 2010 statement^(14)^.

### Participants

The study was conducted in a sample of 9004 participants (age >= 18) representative of the UK adult population with regard to age, gender, income, region and BMI using quota sampling. Inclusion criteria comprised: being over 18 years of age; being able to speak and read English; being UK residents; being willing and able to give informed consent and having access and familiarity with a computer and the Internet. Participants were recruited into the Predictiv, an online experiment platform owned by the Behavioural Insights Team, through Lucid, an online survey panel aggregator. Recruitment of participants was carried out between 3 October 2023 and 11 November 2023. Participants received a small financial reward for taking part in the study.

### Study design

This study was a three-arm randomised controlled trial. Participants were randomised to complete a shopping task in a simulated online supermarket platform under one of the three conditions: (1) HFSS price promotions present and labelled as promotions (‘Usual Practice’, UP: control group); (2) price reductions present on HFSS products but not labelled as a promotion (‘No Communication’, NC) and (3) price promotions removed from HFSS confectionery and snack products (‘No Promotion’, NP).

Participants were equally and individually randomised by Predictiv with allocation concealment. Investigators were unaware and unable to manipulate study parameters after initial study set up. Participants were only aware of the trial arm that they were exposed to and were unaware of other trial arms.

The study was pre-registered on Open Science Framework (https://osf.io/3h4ur/).

Details of the sample size calculation was reported in our pre-registered protocol (https://osf.io/3h4ur/). In short, our power calculation estimated that, given a sample of 9000 participants, three trial arms, adjusting for three comparisons (all intervention conditions were to be compared against the control and to each other) via the Bonferroni correction, we were powered to detect an effect size of 244 calories (Cohen’s d = 0·054), assuming an outcome sd of 4500 calories, with 80 % power. Assumptions involved in the sample size calculation were based on a previous study following similar designs^(15)^.

### Intervention

The study used a simulated online supermarket platform, hosted by the University of Oxford, which emulates a real online supermarket for research purposes relating to food purchasing interventions (www.woodssupermarket.co.uk). The site is populated with approximately 9000 supermarket products that were available to purchase in May 2022, taken from foodDB, a database of food and drinks available for purchase in six UK online supermarkets^(16)^. Products were categorised into a three category hierarchy: Shelf (*n* 461), Aisle (*n* 71) and Department (*n* 5), adapted from the biggest online supermarket in sales in the UK (Tesco.com)^(17)^. The features and functionalities of this simulated online supermarket platform were similar to a real online supermarket, but participants do not spend money or receive their selected items. This experimental online supermarket platform was used in previous studies of various experimental designs^(13,18–20)^. Details of the experimental online supermarket platform were described in our pre-registered protocol and are shown in online supplementary material, Supplemental Appendix Figures S1–S4.

**Figure 1.**
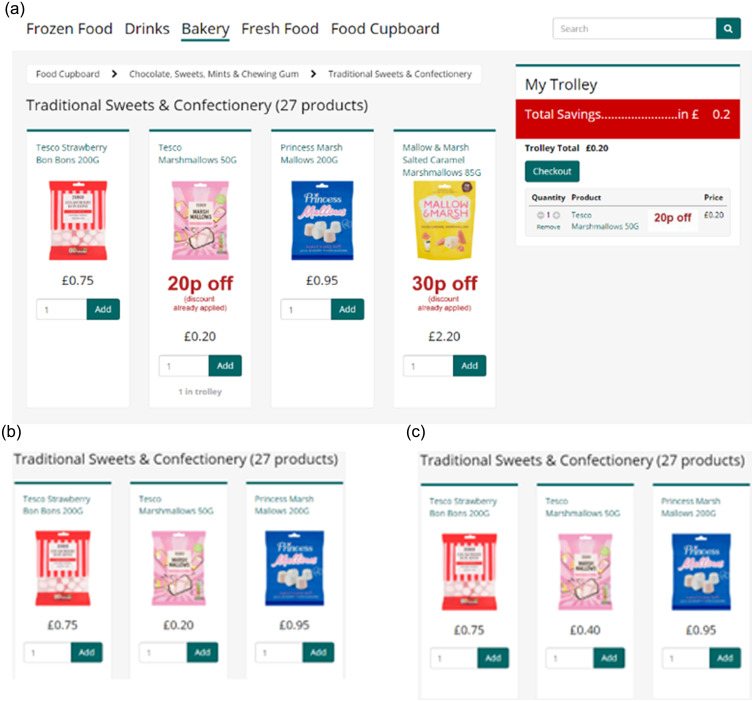
Figure 1 long description. Examples of experimental online supermarket platform layouts for each intervention arm. (a) UP (Usual Practice), (b) NC (No Communication), (c) NP (no promotion).

Participants were randomised to one of the three intervention arms for their shopping task (see Figure 1):

UP: Discounts on selected items in all product categories, including on HFSS products. These discounts were communicated clearly to shoppers.

NC: Discounts on selected items in all product categories, including on HFSS products. However, whilst discounts on non-HFSS products were still communicated to customers through promotion shout-outs, discounts on HFSS products were not communicated.

NP: Discounts on selected non-HFSS products only (clearly communicated to shoppers). HFSS products were not discounted.

HFSS products were defined as products that were classed as HFSS if they were both in-scope of HFSS regulations, according to guidance set out by the Department of Health and Social Care^(21)^, and reached the threshold for HFSS according to the Nutrient Profiling Model^(22)^. Details of classifications of HFSS in this study were described in out pre-registered protocol (https://osf.io/3h4ur/). The number of HFSS and non-HFSS items within each Aisle and Department of the simulated online supermarket can be found in online supplementary material, Supplemental Table S2.

Calculation of the number and size of discounts applied to products was based on purchasing data from a UK supermarket, with details reported in the pre-registered protocol (https://osf.io/3h4ur/). The proportion of products discounted within each product category (e.g. cereals, desserts, coffee, etc.) ranged from 15 % to 44 % (see online supplementary material, Supplemental Table S1 for percentage values). For discounted products, we randomly assigned the products to be discounted to one of four levels, with the extent of these levels of discount varying to reflect differences by product category.

### Procedure

After providing informed consent, participants were directed to Predictiv to complete a baseline questionnaire on demographic characteristics (see pre-registered protocol), including an attention check, and received instructions for a hypothetical food shopping task they would complete as part of the study.

They were then randomised and redirected to Woods simulated online supermarket platform to complete their shopping task. For the shopping task, participants were asked to imagine they were buying food and drinks for themselves and their household for 2 days. They were asked to choose food and drinks that they and their household would want and would be willing to pay for. Participants then answered questions on the number of days they were ordering for, the number of people they were ordering for and whether they usually shop for this many people, to check that they understood the shopping task. No limits on the budget were given to participants for the shopping task. There were no limits on the food categories (e.g. confectionary and snacks) participants could shop from.

Participants then returned to Predictiv for a post-intervention survey on shopping habits and the acceptability of the intervention (measured by asking participants to rate their support for the intervention they were assigned to on a 5-point scale (1 = strongly oppose, 5 = strongly support)).

### Analysis

Data analyses were conducted using RStudio (Version 2025.05.0+496). We reported descriptive statistics (e.g. mean, sd, %) for all outcomes.

#### Data cleaning

Duplicate participants (defined by repeated participant identifiers, IP addresses or browser cookies); participants who discontinued the study and outliers were excluded from the final analysis. Outliers were defined based on the cost of shopping baskets per person in the household. Participants whose basket costs per person in the household were > 1·5 times the interquartile range below quantile 1, or > 1·5 times the interquartile range above quantile 3, were defined as outliers. Outliers (*n* 612) were removed from final analysis.

#### Missing data

Participants (*n* 34) who had missing data on sex, BMI or one outcome measure (proportion of basket that is HFSS items) were treated with listwise deletion due to the small number of such missing data. There were missing data on education (*n* 1227) due to technical errors in the data collection where education data was not recorded correctly and on area-level deprivation (*n* 71) due to participants not inputting required data in correct format. Multiple imputation was used for missing data.

#### Primary analysis

The primary analysis assessed the impact of restricting HFSS price promotions, or restricting the communication of such promotions on total energy (kcal) purchased during the shopping task. The primary outcome was total energy (kcal) placed in the basket, in line with the outcome used in previous work^(13)^. Histograms were used to confirm whether total energy (kcal) in basket was positively skewed. Given this skew, a gamma generalised linear model (GLM) was used to estimate the effects of interventions on total energy (kcal) in basket. We controlled for a list of covariates (see section 2.5.6) in the primary analysis. However, we also conducted *ad hoc* (not pre-specified) sensitivity analyses without controlling for these covariates to assess the robustness of the models.

#### Secondary analysis

Secondary analyses assessed the impact of the interventions on the total spend during the shopping task, as well as participants’ support for such interventions. Secondary outcomes included total cost (£) of the basket and participants’ support for promotion restrictions. Histograms confirmed that total cost (£) of the basket was positively skewed, and support for promotion restriction was normally distributed. For secondary outcomes, a gamma GLM was used to estimate the effects of interventions on total cost of baskets at checkout, and a linear regression was used to estimate the effects of interventions on participants’ support for promotion restrictions.

#### Exploratory analysis

Exploratory outcomes were (1) the number of items in basket at checkout, (2) the proportion of the basket that comprised HFSS items and (3) mean energy density (kcal/100 g) of the basket at checkout. For these analyses, a Poisson GLM was used to assess the impact of the interventions on the number of items in basket at checkout, while linear regressions were conducted to assess the impact of the interventions on the proportion of the basket that comprised HFSS items, and on the mean energy density of basket at checkout. We also conducted a gamma GLM with interaction terms to examine the gender differences in the effects of interventions on total energy (kcal) in basket.

#### Covariates

Covariates, including sex, age, household income, region, area-level deprivation (index of multiple deprivation; IMD decile), education attainment, BMI, ethnic group, urbanicity, supermarket shopping frequency, food shopping responsibility, number of people participants were shopping for (estimated household size), time of day the participant completed their order and day of week the participant completed their order, were controlled for in all analyses.

## Results

### Participant demographics

**Figure 2.**
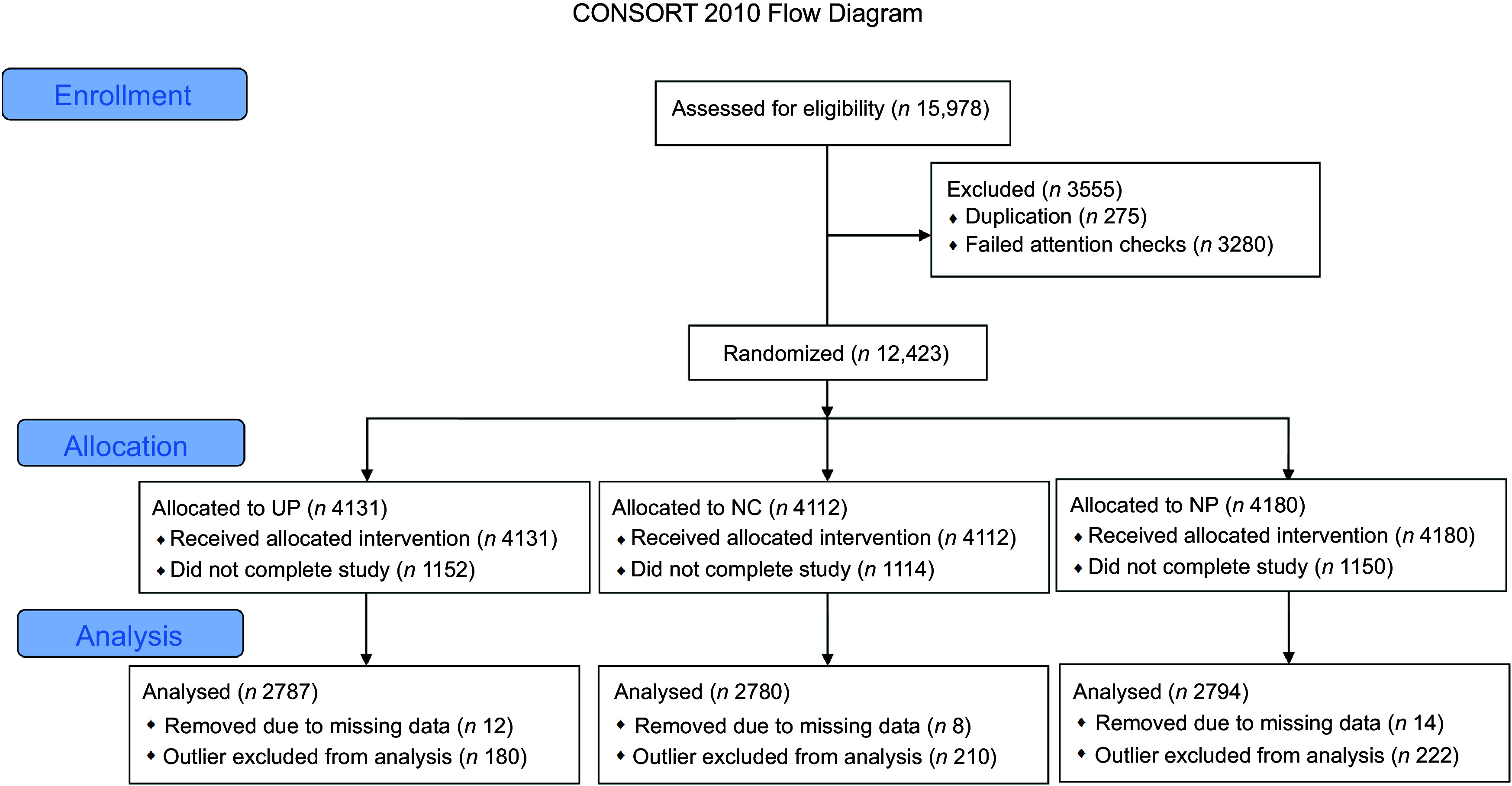
CONSORT flow diagram.

Eligibility was assessed among 15 978 participants, after which 12 423 participants were randomised to one of the three groups, and data were analysed for 8361 participants. Figure 2 shows the participant flow.

Table 1 shows the demographic characteristics for all participants and by groups. In short, over half of the participants were female (55 %), the mean age of participants was 42 years (sd = 15 years), the mean BMI was 26·2 (sd = 6·7) and 81 % of participants were White British. The majority of participants were responsible for grocery shopping (86 %) in their households, and over half of participants (55 %) usually shopped for 2–3 people. There were no significant differences in demographic characteristics between study groups (ANOVA/*χ* ^2^ tests; all *P* > 0·05). Demographic characteristics of participants who discontinued the study (*n* 3416) are shown in online supplementary material, Supplemental Appendix Table S3. Participants who discontinued the study were slightly older (mean = 45 years, sd = 16 years) and had higher BMI (mean = 26·5, sd = 7·7) than those who completed the study. Statistically significant differences were observed across several socio-demographic variables, including income, education, ethnicity and responsibility for household shopping. However, these were very small effects, and given the large sample size, these are likely to represent relatively minor differences in sample composition.

**Table 1.** Characteristics of trial participants and their selected baskets by study condition Table 1 long description.

		UP (*n* 2787)			NC (*n* 2780)			NP (*n* 2794)			Total (*n* 8361)		
		Mean	sd	%	Mean	sd	%	Mean	sd	%	Mean	sd	%
Age	Mean (sd)	42	15		42	15		42	15		42	15	
Gender	Male			44			45			46			45
	Female			56			55			54			55
Income	< £20 000			25			25			26			25
	£20 000–£39 999			34			31			33			33
	£40 000–£59 999			22			23			23			23
	≥ £60 000			19			21			19			19
Region	South and East			30			30			29			30
	North			27			26			26			26
	Midlands			18			18			18			18
	London			12			13			13			13
	Scotland, NI, Wales			13			14			14			13
BMI	Mean (sd)	26·1	6·5		26·3	6·8		26·3	6·9		26·2	6·8	
Ethnicity	White UK			82			81			82			82
	Asian or Asian British			6			6			6			6
	Black			4			6			5			5
	White Other			5			5			4			5
	Mixed			2			2			2			2
	Other			1			1			1			1
Education	Less than high school			4			3			4			4
	High school completed			21			20			21			21
	University degree			37			37			39			38
	None of the above			23			25			22			23
	Missing			15			15			14			15
Index of Multiple Deprivation	1st decile			6			6			6			6
	2nd decile			7			6			6			7
	3rd decile			7			9			8			8
	4th decile			20			20			18			19
	5th decile			13			14			16			14
	6th decile			11			12			11			11
	7th decile			11			10			11			11
	8th decile			8			8			7			8
	9th decile			10			10			9			10
	10th decile			6			5			5			5
	Missing			1			1			1			1
Number of people in household	1			14			15			16			15
	2			34			32			32			33
	3			23			22			21			22
	> 3			29			31			31			31
Responsible for shopping	Yes			86			86			86			86
	No			14			14			14			14
Total energy selected (kcal)	Mean (sd)	10 280	9684		10 081	9649		10 110	10 266		10 157	9869	
Total cost of basket (£GBP)	Mean (sd)	18·13	13·77		18·18	14·24		18·11	13·65		18·14	13·89	
Number of items in basket (*n*)	Mean (sd)	11·4	9·2		11·3	9·1		11·1	8·7		11·3	9·0	
Proportion of items in basket that was on promotion	%			30			31			30			30
Proportion of basket that was HFSS items	%			23			22			23			23
Energy density of basket (kcal/100 g)	Mean (sd)	2·06	1·74		2·03	1·59		2·18	7·79		2·09	4·70	
Policy support^*^	Mean (sd)	2·54	1·03		2·26	1·03		2·04	1·22		2·28	1·11	

* Values from 1 (‘strongly oppose’) to 5 (‘strongly support’).

### Primary outcome

**Figure 3.**
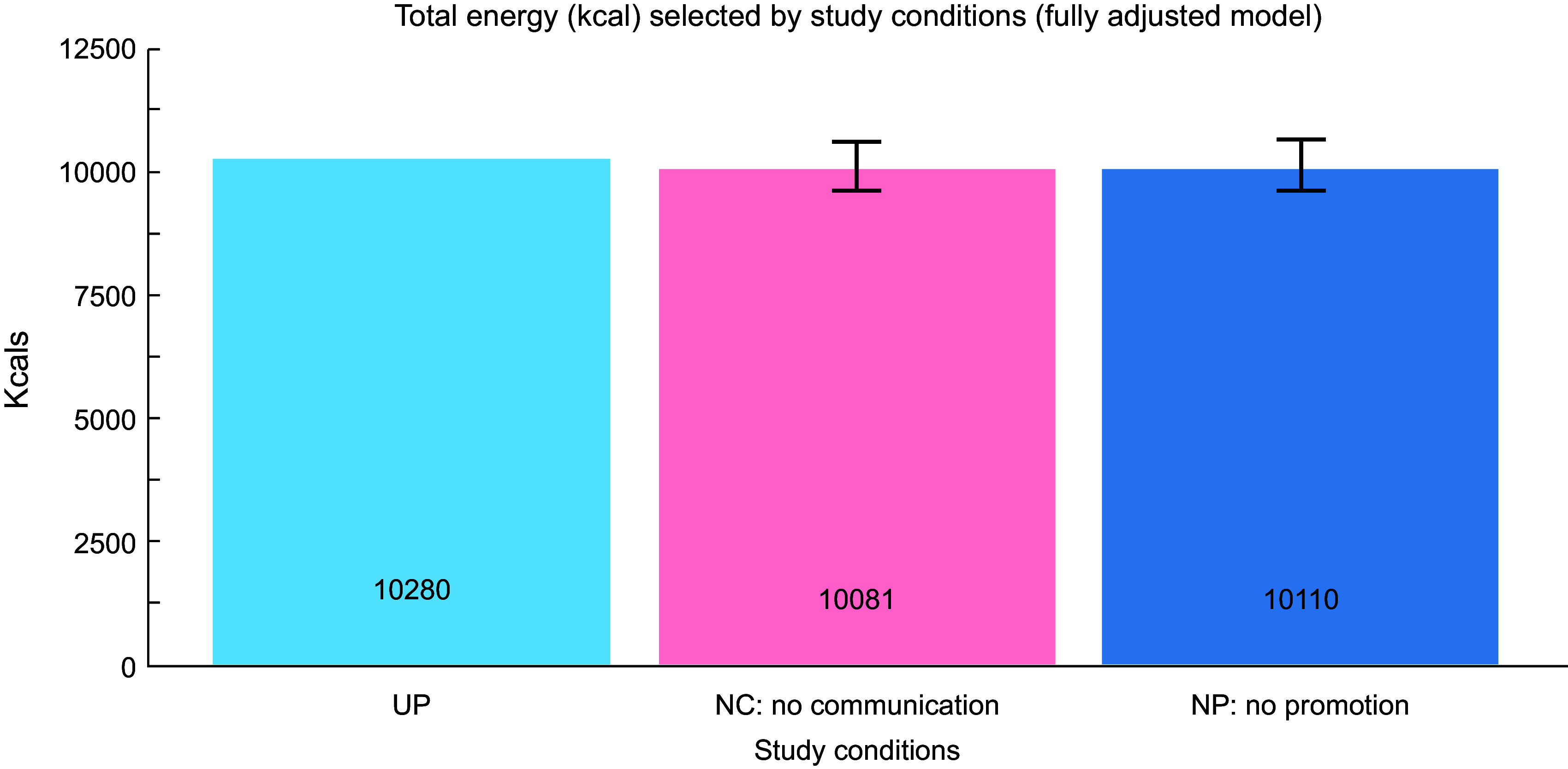
Differences in total energy selected by study conditions.

Table 2 shows the results for primary outcome. On average, participants in the control group (UP) selected 10 280 (sd = 9684) kcal, participants in NC selected 10 081 (sd = 9649) kcal and participants in NP selected 10 110 (sd = 10 266) kcal in their shopping basket. Although individuals in both NC (estimates = –201 kcal, 95 % CI: (–650 kcal, 269 kcal), *P* = 0·40) and NP (estimates = –193 kcal, 95 % CI: (–648 kcal, 285 kcal), *P* = 0·20) selected fewer calories compared with UP, neither of the differences were statistically significant, controlling for covariates. There was no significant difference in the total energy in basket between NP and NC (estimates = –9 kcal, 95 % CI: (–449 kcal, 489 kcal), *P* = 0·97), controlling for covariates (see Figure 3). We also conducted a sensitivity analysis to examine the intervention effects without controlling for covariates. Results remain the same: participants in neither NC (estimates = –204 kcal, 95 % CI: (–695 kcal, 313 kcal), *P* = 0·44) nor NP (estimates = –204 kcal, 95 % CI: (–695 kcal, 313 kcal), *P* = 0·52) selected significantly fewer calories than participants in UP.

**Table 2.** Comparison of primary, secondary and exploratory outcomes between trial groups Table 2 long description.

	Coefficient	95 % CI	Coefficient *P*
**Primary outcome**			
Total energy selected (kcal)			
NC *v.* UP	−201 kcal	−650 kcal, 269 kcal	0·40
NP *v.* UP	−193 kcal	−648 kcal, 285 kcal	0·42
NP *v.* NC	−9 kcal	−449 kcal, 489 kcal	0·97
**Secondary outcomes**			
Total cost of basket (£)			
NC *v.* UP	-£0·05	−£0·68, £0·59	0·87
NP *v.* UP	£0·16	−£0·47, £0·82	0·62
NP *v.* NC	£0·22	−£0·42, £0·88	0·51
Policy support			
**NC** ***v.*** **UP**	**−0·28**	**−0·34, −0·23**	**< 0·001**
**NP** ***v.*** **UP**	**−0·51**	**−0·56, −0·45**	**< 0·001**
**NP** ***v.*** **NC**	**−0·22**	**−0·28, −0·17**	**< 0·001**
**Exploratory outcomes**			
Number of items in basket (% fewer items)			
NC *v.* UP	−1 %	−3 %, 1 %	0·20
**NP** ***v**.* **UP**	**−0·2 %**	**−4 %, −1 %**	**0·003**
NP *v.* NC	−1 %	−3 %, 0 %	0·081
Proportion of basket that was HFSS			
NC *v.* UP	−1 pp	−2 pp, 0 pp	0·19
NP *v.* UP	0 pp	−2 pp, 1 pp	0·60
NP *v.* NC	1 pp	−1 pp, 2 pp	0·43
Energy density of basket			
NC *v.* UP	5 kcal/100 g	−30 kcal/100 g, 20 kcal/100 g	0·70
NP *v.* UP	12 kcal/100 g	−13 kcal/100 g, 36 kcal/100 g	0·36
NP *v.* NC	17 kcal/100 g	−8 kcal/100 g, 41 kcal/100 g	0·19

Coefficients of the models are presented as adjusted mean differences in the original units of the outcomes (kcal, £, kcal/100 g) with corresponding 95 % CI for outcomes total energy selected, total cost of basket, number of items in basket, proportion of basket that was HFSS and energy density of basket. pp, percentage points. See online supplementary material, Supplemental Table S4–S9 for full GLM model output. The reference group is the second condition listed in each row description. Comparisons shown in bold are statistically significant.

### Secondary outcomes

The mean cost of baskets for those in UP was £18·13 (sd = 13·77). There were no significant differences in the total cost of basket between study groups, controlling for demographic characteristics (see Table 2).

**Figure 4.**
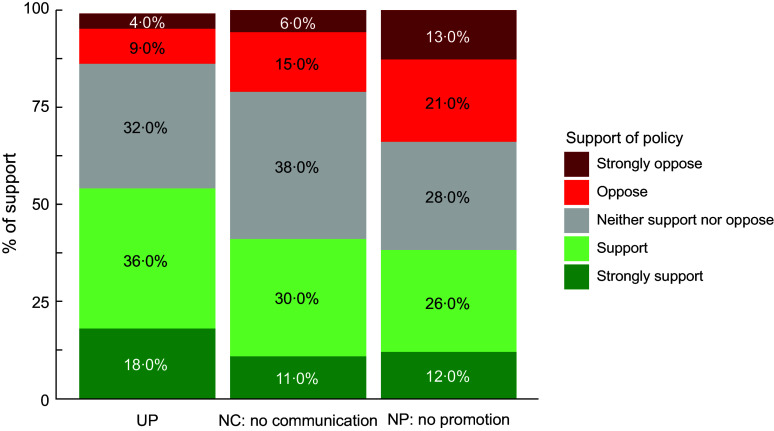
Policy support, as rated by those assigned to that study condition.

Figure 4 shows the proportion of participants expressing support or opposition for the policy they were exposed to. The mean (sd) scores for policy support for each arm are shown in Table 1. Support for restricting promotions from HFSS items completely was significantly lower than that for restricting communications only (*P* < 0·001). However, support for having promotions for HFSS items but restricting communications and restricting promotions for HFSS items completely were both significantly lower than support for usual practice (having clearly communicated promotions; both *P* < 0·001). These results are shown in Table 2.

### Exploratory outcomes

Those in UP selected an average of 11·4 items (sd = 9·2). Those in the group without promotion for HFSS items (NP) had significantly fewer items in their baskets (2 % reduction, approximately 0·2 fewer overall items per person) compared with the UP group (*P* = 0·003, 95 % CI: (–0·04, –0·01)). However, there was no evidence of differences in the number of items in baskets between the NC and the UP group or between NC and NP groups. There was no evidence of significant differences in the proportion of the basket that was HFSS, or in energy density (kcal/100 g) by study groups (see Table 2). There was no robust evidence suggestive of gender differences in the effects of interventions on total energy (kcal) in basket (see online supplementary material, Supplemental Appendix Table S10).

## Discussion

This randomised controlled trial aimed to test the impact of restricting price promotions for HFSS items, or just restricting communications of price promotions for HFSS items (keeping the price reductions), on purchasing behaviours. We found no significant differences in total energy (kcal) in basket, price of basket, the proportion of basket that was HFSS or the mean energy density of basket between study groups, after controlling for demographic factors. Although we did find that participants in the group with no promotions for HFSS items had significantly fewer items in their baskets, compared with the control group (where both promotions and communications were presented), this effect was very small.

This study expanded on the existing literature on the impact of restricting price promotions on purchasing behaviour and provided novel experimental evidence on whether restricting communications regarding price promotions could influence purchasing behaviour. This study was conducted with a large UK-representative sample, where participants were able to select food and drink items that were available in major supermarkets recently, displayed in similar category hierarchy and with their usual prices. Although we designed the simulated online supermarket to look and function as naturalistically as possible, there are some key limitations to this method. Participants did not spend money or receive any items from the shopping task, or have any limitation on the budget for the shopping task. Participants therefore may be less concerned about costs when selecting items in this shopping task, in contrast to usual shopping, where price is often identified as one of the most important considerations^(23,24)^. This may also have made the price and price promotion manipulations less salient than they may be in other contexts. Although we controlled for the number of people the participants usually shop for, we did not collect data on household composition (e.g. number of children), and we acknowledge that this may not account fully for household consumption needs^(25,26)^.

Nevertheless, a previous study using similar methods found that when restricting price promotions for HFSS items, the total energy in basket was significantly reduced (∼10 % lower), compared with control^(13)^. However, we did not observe similar results in our study. This might be due to differences in the shopping tasks used. In the previous study, participants were asked to buy snacks for a night in with four friends and were asked to choose items in four categories: ‘Confectionery’; ‘Biscuits and crackers’; ‘Crisps, nuts and snacking fruit’ and ‘Cakes and tarts’, with a budget of between £5 and £10. These four targeted categories traditionally consist of a high proportion of HFSS items^(27)^. In our study, however, participants were asked to shop for their household for two days with no limits on the budget or direction towards HFSS categories. In this previous study, 42 % of items placed in shopping baskets had price promotions in the ‘promotion’ condition, while in our study, an average of 30 % of items in baskets in the UP group had price promotions. It is possible that the effect of restricting price promotions (and communication of promotions) was reduced when using a broader shopping task rather than a focus on food categories with large proportions of HFSS items (as in Luick *et al.*), given the latter may increase exposure to the study manipulations. This may indicate that the impact of restricting TPR, or the communication of these promotions, on HFSS foods may be more prominent in certain scenarios (e.g. with predominantly discretionary purchases) than others (e.g. weekly grocery shop).

An analysis of take-home food and drink purchases in the UK in 2019 revealed that the median calories purchased per person per week was 10 220 kcal for households who more frequently shopped for groceries online, with HFSS items accounting for approximately 38 % of total items purchased^(28)^. In our study, households purchased a median of 3141 kcal per person over 2 days, which echoes the median energy purchased by UK households per week. However, the proportion of HFSS items in the baskets appeared to be lower in our study (22–23 % of total items selected) compared with previous real life observations^(25)^. It is possible that, as HFSS foods are often discretionary items^(29)^, they were overlooked when participants were asked to shop for food and drinks in general in an experimental task. The relatively low proportion of HFSS items selected in our study may also reduce any potential intervention effect.

When we explored the support for different interventions in the study, we found that restricting promotions entirely had the least support from participants (38 %). Although restricting communications regarding discounts on HFSS items had significantly less support (41 %) compared with the control group (54 %), it had more support than restricting promotions completely. This echoes evidence from a previous study where the majority of participants were in support of legislations but are concerned about affordability^(26)^. This suggests that when considering public policies involving restrictions on price promotions, restricting communications while allowing price reductions might be a potential compromise – maintaining food affordability and likely garnering greater public support. However, the current study did not find this impacted on consumer behaviour, which could suggest that this may not be effective at improving public health. Given this study focused on online supermarket shopping and hypothetical purchases, the impact of restricting communications of discounts should also be explored in alternative settings before robust conclusions can be drawn.

Although we did not find significant effects on purchasing behaviours through restricting price promotions (or related communications) on HFSS foods, considering the limitations of this study, we are yet cautious to conclude that these interventions are ineffective. A hypothesised mechanism for price promotions to influence consumption is that price promotions increase the volume of products purchased, which may lead to increased consumption^(7)^. Existing literature does point to a relationship between price promotion and stockpiling behaviours^(7,11)^, stockpiling and food and drink consumption^(30)^ and increased consumption for certain food and drinks (e.g. crisps) following promotion^(31)^. This pathway through stockpiling, however, may not have occurred in our study because our shopping task was for participants to shop for groceries to cater for over 2 days, thus the anticipated consumption period was over only 2 days. Future studies to investigate the impact of restricting price promotions (or communication of) may avoid this issue by examining repeated shopping tasks or lengthening the observation period. Moreover, using more naturalistic settings including enabling payment or delivery procedures for the shopping task for an online experiment, or conducting field trials in shopper laboratories or actual supermarkets, could overcome some of these limitations. Furthermore, studies where participants take home their selected items should also measure the rate and volume of consumption, to examine the impact of these interventions on speed of consumption and time to next purchase.

### Conclusions

This study did not find significant effects of restricting communications regarding temporary price promotions, or restricting price promotions entirely, on purchasing behaviours in a simulated online supermarket. However, only restricting communications received better public support than restricting price promotions entirely. Further studies on price-based interventions, especially requiring actual payments, are needed to provide robust policy recommendations.

## Supporting information

10.1017/S1368980026103073.sm001 Jia et al. supplementary material Jia et al. supplementary material

## Data Availability

The datasets used and/or analysed during the current study are available from the corresponding author on reasonable request.

## Supplementary material

For supplementary material accompanying this paper visit https://doi.org/10.1017/S1368980026103073

## Figure 1. Long description

Panel A: A screenshot of an online supermarket platform showing the usual practice layout. The page displays various traditional sweets and confectionery items, including Tesco Strawberry Bon Bons, Tesco Marshmallows, Princess Marsh Mallows, and Mallow & Marsh Salted Caramel Marshmallows. Discounts are applied to some items, and a trolley on the right side shows the total savings and items added. Panel B: A screenshot of an online supermarket platform showing a layout with no communication. The page displays the same items as in Panel A, but without any discounts or special offers. Panel C: A screenshot of an online supermarket platform showing a layout with no promotion. The page displays the same items as in Panel A and B, with further reduced prices for some items.

Navigate back to Figure 1..

## Table 1. Long description

The table compares demographic characteristics and basket selections of trial participants across three study conditions: UP, NC, and NP. It includes data on age, gender, income, region, BMI, ethnicity, education, index of multiple deprivation, number of people in the household, and responsibility for shopping. The table also provides information on total energy selected, total cost of the basket, number of items in the basket, proportion of items on promotion, proportion of basket that was HFSS items, energy density of the basket, and policy support. Each row represents a different characteristic or variable, with columns showing the mean, standard deviation, and percentage for each study condition. The table has 42 rows and 15 columns, with detailed values for each characteristic across the three conditions.

Navigate back to Table 1..

## Table 2. Long description

The table presents a comparison of primary, secondary, and exploratory outcomes between three trial groups: NC, NP, and UP. It consists of 12 rows and 5 columns, including headers. The columns are labeled Coefficient, 95% CI, and Coefficient P. The rows are categorized into Primary outcome, Secondary outcomes, Policy support, Exploratory outcomes, and further subcategories. Each row provides specific data points for the comparisons between NC v. UP, NP v. UP, and NP v. NC. The table includes values for total energy selected in kilocalories, total cost of the basket in pounds, policy support, number of items in the basket as a percentage, proportion of the basket that was HFSS, and energy density of the basket in kilocalories per 100 grams. Notable trends include the lack of statistically significant differences in total energy selected between the groups, as indicated by the P values greater than 0.05. The table also shows variations in the total cost of the basket, policy support, number of items, and energy density among the groups.

Navigate back to Table 2..
