# Supplementary material for: The impact of restricting price promotions on foods high in fat, sugar and salt on purchasing behaviours: a randomised controlled trial in a simulated online supermarket in the UK
Source: Public Health Nutr. 2026 Jul 9;29(1):e156. doi: 10.1017/S1368980026103073 (PMC13434572; doi:10.1017/S1368980026103073)
Supplement: Supplementary file 1 [file S1368980026103073sup001.docx]

**Supplementary Appendix**

[Figure S1 Example of category dropdown menu	2](#_Toc218853596)

[Figure S2 Example of a shelf when participants were browsing for products	2](#_Toc218853597)

[Figure S3 Example of using the search function	3](#_Toc218853598)

[Figure S4 Example of the page when participants add (an) item(s) into their basket	3](#_Toc218853599)

[Table S1. Proportion of products discounted within each product category	4](#_Toc218853600)

[Table S2 Number of HFSS and non-HFSS items within each Aisle and Department of the simulated online supermarket	5](#_Toc218853601)

[Table S3. Demographic characteristics of participants who discontinued the study	8](#_Toc218853602)

[Table S4. Full GLM model output for primary outcome: total energy selected	9](#_Toc218853603)

[Table S5. Full GLM model output for secondary outcome: total price of basket (£)	11](#_Toc218853604)

[Table S6. Full GLM model output for secondary outcome: policy support	12](#_Toc218853605)

[Table S7. Full GLM model output for exploratory outcome: number of items in basket	14](#_Toc218853606)

[Table S8. Full GLM model output for exploratory outcome: proportion of basket that is HFSS items	15](#_Toc218853607)

[Table S9. Full GLM model output for exploratory outcome: calorie density of basket	16](#_Toc218853608)

[Table S10. Full GLM model with interaction terms output for exploratory outcome: moderating effects of gender on the effects of intervention on total energy (kcal) in basket	18](#_Toc218853609)

Figure S1 Example of category dropdown menu

Figure S2 Example of a shelf when participants were browsing for products

Figure S3 Example of using the search function

Figure S4 Example of the page when participants add (an) item(s) into their basket

Table S1. Proportion of products discounted within each product category

| **Product category (shown as Aisle)** | **Proportion of products discounted (%)** | | |
| --- | --- | --- | --- |
|  | **UP** | **NC** | **NP** |
| Adult Soft Drinks & Mixers | 34 | 34 | 9 |
| Alcohol Free & Low Alcohol Drinks | 42 | 42 | 42 |
| Bakery Free From | 24 | 24 | 7 |
| Beer & Cider | 38 | 38 | 38 |
| Biscuits & Cereal Bars | 34 | 34 | 1 |
| Bread & Rolls | 25 | 25 | 25 |
| Cakes, Cake Bars, Slices & Pies | 23 | 23 | 0 |
| Cereals | 34 | 34 | 23 |
| Cheese | 30 | 30 | 29 |
| Chilled Desserts | 25 | 25 | 1 |
| Chilled Fish & Seafood | 17 | 17 | 17 |
| Chilled Soup, Sandwiches & Salad Pots | 20 | 20 | 17 |
| Chilled Vegetarian & Vegan | 27 | 27 | 18 |
| Chocolate, Sweets, Mints & Chewing Gum | 37 | 37 | 0 |
| Coffee | 41 | 41 | 41 |
| Cooked Meats, Antipasti & Dips | 26 | 26 | 7 |
| Cooking Ingredients | 33 | 33 | 32 |
| Cooking Sauces, Meal Kits & Sides | 34 | 34 | 11 |
| Crackers & Crispbreads | 32 | 32 | 14 |
| Crisps, Snacks, Nuts & Popcorn | 35 | 35 | 3 |
| Croissants, Brioche & Pastries | 25 | 25 | 0 |
| Crumpets, Muffins & Pancakes | 22 | 22 | 6 |
| Dairy Free & Dairy Alternatives | 30 | 30 | 30 |
| Desserts | 24 | 24 | 13 |
| Doughnuts, Cookies & Muffins | 23 | 23 | 3 |
| Dried Fruit, Nuts, Nutrient Powders & Seeds | 22 | 22 | 22 |
| Dried Pasta, Rice, Noodles & Cous Cous | 31 | 31 | 31 |
| Fizzy & Soft Drinks | 33 | 33 | 17 |
| Fresh Fruit | 20 | 20 | 19 |
| Fresh Meat & Poultry | 22 | 22 | 22 |
| Fresh Pizza, Pasta & Garlic Bread | 31 | 31 | 27 |
| Fresh Salad, Coleslaw & Sandwich Fillers | 22 | 22 | 22 |
| Fresh Vegetables | 19 | 19 | 17 |
| From Our Bakery | 20 | 20 | 7 |
| Frozen Breakfast, Fruit & Pastry | 31 | 31 | 12 |
| Frozen Chips, Onion Rings, Potatoes & Rice | 23 | 23 | 21 |
| Frozen Fish & Seafood | 21 | 21 | 21 |
| Frozen Free From | 27 | 27 | 4 |
| Frozen Meat & Poultry | 15 | 15 | 15 |
| Frozen Party Food & Sausage Rolls | 29 | 29 | 4 |
| Frozen Pies | 28 | 28 | 22 |
| Frozen Pizza & Garlic Bread | 24 | 24 | 14 |
| Frozen Ready Meals | 30 | 30 | 28 |
| Frozen Vegetables & Herbs | 29 | 29 | 29 |
| Frozen Vegetarian & Vegan | 35 | 35 | 31 |
| Frozen World Foods & Halal | 29 | 29 | 14 |
| Frozen Yorkshire Puddings & Stuffing | 29 | 29 | 14 |
| Home Baking | 25 | 25 | 23 |
| Hot Chocolate & Malted Drinks | 40 | 40 | 37 |
| Ice Cream, Ice Lollies & Frozen Desserts | 38 | 38 | 0 |
| Jams, Sweet & Savoury Spreads | 31 | 31 | 4 |
| Juice & Smoothies | 28 | 28 | 15 |
| Juices & Smoothies | 29 | 29 | 27 |
| Kids Drinks | 31 | 31 | 19 |
| Milk, Butter & Eggs | 31 | 31 | 31 |
| Milkshake | 28 | 28 | 28 |
| On The Go Drinks | 28 | 28 | 22 |
| Pies, Pasties, Quiches & Snacking | 40 | 40 | 6 |
| Ready Meals | 27 | 27 | 25 |
| Ready To Drink Wine & Spirits | 25 | 25 | 25 |
| Spirits | 25 | 25 | 25 |
| Sports, Energy & Wellness Drinks | 33 | 33 | 24 |
| Squash & Cordial | 39 | 39 | 39 |
| Table Sauces, Olives, Pickles & Chutneys | 25 | 25 | 25 |
| Tea | 44 | 44 | 44 |
| Teacakes, Fruit Loaves & Scones | 25 | 25 | 25 |
| Tins, Cans & Packets | 19 | 19 | 15 |
| Water | 35 | 35 | 35 |
| Wine | 31 | 31 | 31 |
| Wraps, Pittas, Naan & Thins | 21 | 21 | 21 |
| Yoghurts | 28 | 28 | 24 |

Table S2 Number of HFSS and non-HFSS items, and the proportion of HFSS on promotion (UP/NS) within each Aisle and Department of the simulated online supermarket

| **Department** | **Aisle** | **HFSS** | **Non-HFSS** | **Proportion of HFSS on promotion (UP/NS)** |
| --- | --- | --- | --- | --- |
| Bakery | Bakery Free From | 42 | 30 | 29 |
| Bakery | Bread & Rolls | 0 | 118 | NA |
| Bakery | Cakes, Cake Bars, Slices & Pies | 251 | 1 | 23 |
| Bakery | Croissants, Brioche & Pastries | 32 | 0 | 25 |
| Bakery | Crumpets, Muffins & Pancakes | 13 | 5 | 23 |
| Bakery | Doughnuts, Cookies & Muffins | 34 | 1 | 21 |
| Bakery | From Our Bakery | 66 | 24 | 18 |
| Bakery | Teacakes, Fruit Loaves & Scones | 2 | 2 | 0 |
| Bakery | Wraps, Pittas, Naan & Thins | 0 | 14 | NA |
| Drinks | Adult Soft Drinks & Mixers | 47 | 18 | 34 |
| Drinks | Alcohol Free & Low Alcohol Drinks | 0 | 33 | NA |
| Drinks | Beer & Cider | 0 | 104 | NA |
| Drinks | Coffee | 0 | 81 | NA |
| Drinks | Fizzy & Soft Drinks | 24 | 52 | 38 |
| Drinks | Hot Chocolate & Malted Drinks | 0 | 35 | NA |
| Drinks | Juices & Smoothies | 5 | 112 | 40 |
| Drinks | Kids Drinks | 13 | 29 | 38 |
| Drinks | Milkshake | 3 | 65 | 0 |
| Drinks | On The Go Drinks | 25 | 47 | 16 |
| Drinks | Ready To Drink Wine & Spirits | 0 | 4 | NA |
| Drinks | Spirits | 0 | 4 | NA |
| Drinks | Sports, Energy & Wellness Drinks | 14 | 35 | 29 |
| Drinks | Squash & Cordial | 1 | 35 | 0 |
| Drinks | Tea | 1 | 42 | 0 |
| Drinks | Water | 0 | 23 | NA |
| Drinks | Wine | 0 | 13 | NA |
| Food Cupboard | Biscuits & Cereal Bars | 275 | 7 | 34 |
| Food Cupboard | Cereals | 131 | 209 | 29 |
| Food Cupboard | Chocolate, Sweets, Mints & Chewing Gum | 472 | 1 | 35 |
| Food Cupboard | Cooking Ingredients | 5 | 291 | 40 |
| Food Cupboard | Cooking Sauces, Meal Kits & Sides | 373 | 154 | 32 |
| Food Cupboard | Crackers & Crispbreads | 43 | 20 | 26 |
| Food Cupboard | Crisps, Snacks, Nuts & Popcorn | 272 | 39 | 36 |
| Food Cupboard | Desserts | 54 | 72 | 24 |
| Food Cupboard | Dried Fruit, Nuts, Nutrient Powders & Seeds | 0 | 158 | NA |
| Food Cupboard | Dried Pasta, Rice, Noodles & Cous Cous | 0 | 157 | NA |
| Food Cupboard | Home Baking | 38 | 183 | 13 |
| Food Cupboard | Jams, Sweet & Savoury Spreads | 98 | 10 | 31 |
| Food Cupboard | Table Sauces, Olives, Pickles & Chutneys | 0 | 366 | NA |
| Food Cupboard | Tins, Cans & Packets | 72 | 381 | 26 |
| Fresh Food | Cheese | 19 | 201 | 16 |
| Fresh Food | Chilled Desserts | 239 | 14 | 25 |
| Fresh Food | Chilled Fish & Seafood | 0 | 90 | NA |
| Fresh Food | Chilled Soup, Sandwiches & Salad Pots | 20 | 118 | 25 |
| Fresh Food | Chilled Vegetarian & Vegan | 64 | 78 | 20 |
| Fresh Food | Cooked Meats, Antipasti & Dips | 252 | 127 | 28 |
| Fresh Food | Dairy Free & Dairy Alternatives | 0 | 193 | NA |
| Fresh Food | Fresh Fruit | 0 | 147 | NA |
| Fresh Food | Fresh Meat & Poultry | 0 | 259 | NA |
| Fresh Food | Fresh Pizza, Pasta & Garlic Bread | 11 | 40 | 18 |
| Fresh Food | Fresh Salad, Coleslaw & Sandwich Fillers | 0 | 200 | NA |
| Fresh Food | Fresh Vegetables | 0 | 156 | NA |
| Fresh Food | Juice & Smoothies | 45 | 44 | 27 |
| Fresh Food | Milk, Butter & Eggs | 0 | 232 | NA |
| Fresh Food | Pies, Pasties, Quiches & Snacking | 136 | 27 | 40 |
| Fresh Food | Ready Meals | 45 | 661 | 31 |
| Fresh Food | Yoghurts | 73 | 255 | 18 |
| Frozen Food | Frozen Breakfast, Fruit & Pastry | 6 | 10 | 50 |
| Frozen Food | Frozen Chips, Onion Rings, Potatoes & Rice | 4 | 48 | 25 |
| Frozen Food | Frozen Fish & Seafood | 0 | 63 | NA |
| Frozen Food | Frozen Free From | 19 | 7 | 32 |
| Frozen Food | Frozen Meat & Poultry | 10 | 88 | 0 |
| Frozen Food | Frozen Party Food & Sausage Rolls | 20 | 8 | 35 |
| Frozen Food | Frozen Pies | 7 | 11 | 14 |
| Frozen Food | Frozen Pizza & Garlic Bread | 18 | 19 | 22 |
| Frozen Food | Frozen Ready Meals | 3 | 84 | 67 |
| Frozen Food | Frozen Vegetables & Herbs | 0 | 21 | NA |
| Frozen Food | Frozen Vegetarian & Vegan | 9 | 56 | 33 |
| Frozen Food | Frozen World Foods & Halal | 7 | 7 | 29 |
| Frozen Food | Frozen Yorkshire Puddings & Stuffing | 1 | 6 | 100 |
| Frozen Food | Ice Cream, Ice Lollies & Frozen Desserts | 209 | 7 | 38 |

Table S3. Demographic characteristics of participants who discontinued the study

|  |  | **Participants who discontinued the study (n= 3416)** | **Participants who completed the study (n=8361)** | **Statistics** |
| --- | --- | --- | --- | --- |
| Age | Mean (SD) | 45 (16) | 42 (15) | t=10.2, p<0.001 |
| Gender | Male | 43% | 45% | χ²(2)=4.6, p=0.10 |
|  | Female | 56% | 55% |  |
|  | Other or missing | < 1% | - |  |
| Income | < £20,000 | 30% | 25% | χ²(3)=35.4, p<0.001 |
|  | £20,000-£39,999 | 33% | 33% |  |
|  | £40,000-£59,999 | 20% | 23% |  |
|  | ≥£60,000 | 17% | 19% |  |
| Region | South and East | 30% | 30% | χ²(4)=18.9, p=0.001 |
|  | North | 27% | 26% |  |
|  | Midlands | 18% | 18% |  |
|  | London | 10% | 13% |  |
|  | Scotland, NI, Wales | 15% | 13% |  |
| BMI | Mean (SD) | 26.54 (7.74)* | 26.2 (6.8) | t=2.6, p=0.009 |
| Ethnicity | White UK | 85% | 82% | χ²(5)=29.7, p<0.001 |
|  | Asian or Asian British | 4% | 6% |  |
|  | Black | 4% | 5% |  |
|  | White Other | 4% | 5% |  |
|  | Mixed | 2% | 2% |  |
|  | Other | 1% | 1% |  |
| Education | Less than high school | 5% | 4% | χ²(4)=67.8, p<0.001 |
|  | High school completed | 25% | 21% |  |
|  | University degree | 36% | 38% |  |
|  | None of the above | 18% | 23% |  |
|  | Missing | 15% | 15% |  |
| Index of Multiple Deprivation | 1st decile | 7% | 6% | χ²(10)=7.9, p=0.64 |
|  | 2nd decile | 7% | 7% |  |
|  | 3rd decile | 8% | 8% |  |
|  | 4th decile | 20% | 19% |  |
|  | 5th decile | 14% | 14% |  |
|  | 6th decile | 11% | 11% |  |
|  | 7th decile | 11% | 11% |  |
|  | 8th decile | 7% | 8% |  |
|  | 9th decile | 9% | 10% |  |
|  | 10th decile | 5% | 5% |  |
|  | Missing | 1% | 1% |  |
| Number of people in household | 1 | 17% | 15% | χ²(3)=16.4, p=0.001 |
|  | 2 | 34% | 33% |  |
|  | 3 | 21% | 22% |  |
|  | >3 | 28% | 31% |  |
| Responsible for shopping | Yes | 82% | 86% | χ²(1)=31.2, p<0.001 |
|  | No | 18% | 14% |  |

*Note that 17 rows or <1% of the data was missing, so excluded from this calculation.

Table S4. Full GLM model output for primary outcome: total energy selected

| **Primary Outcome: Total energy selected** | **Multiple Imputation Estimates (95%CI)** |
| --- | --- |
| (Intercept) | 3428.71** (2898.33,4056.14) |
| NC vs UP | 0.98 (0.94,1.03) |
| NP vs UP | 0.98 (0.94,1.03) |
| NP vs NC | 1 (0.96,1.05) |
| Gender: Women vs Men | 1.21** (1.16,1.26) |
| Gender: Other vs Men | 1.15 (0.83,1.59) |
| Age: 25-34 vs 18-24 | 1.01 (0.93,1.08) |
| Age: 35-44 vs 18-34 | 1.06 (0.98,1.14) |
| Age: 45-54 vs 18-34 | 1.08* (1.01,1.17) |
| Age: 55-64 vs 18-34 | 1.26** (1.17,1.37) |
| Age: 65 and over vs 18-34 | 1.2** (1.1,1.3) |
| Income: £20,000- £39,999 vs under £20,000 | 1.01 (0.96,1.06) |
| Income: £40,000 - £59,999 vs under £20,000 | 0.98 (0.93,1.04) |
| Income: Over £60,000 vs under £20,000 | 1 (0.94,1.07) |
| Location: North vs London | 1.06 (0.99,1.14) |
| Location: South and East vs London | 1.08* (1,1.16) |
| Location: Midland vs London | 1.07+ (0.99,1.16) |
| Location: Wales, Scotland and Northern Ireland vs London | 1.03 (0.93,1.16) |
| Urban: Rural vs Urban | 1.08** (1.02,1.15) |
| Urban: Suburban vs Urban | 1.06* (1.01,1.12) |
| Ethnicity: White Other vs White British | 1.03 (0.94,1.12) |
| Ethnicity: Asian or Asian British vs White British | 0.99 (0.88,1.11) |
| Ethnicity: Black vs White British | 1.15** (1.04,1.27) |
| Ethnicity: Mixed vs White British | 1.11 (0.97,1.27) |
| Ethnicity: Other vs White British | 0.9 (0.72,1.12) |
| Shopping frequency: Once a week vs More than once a week | 1 (0.94,1.07) |
| Shopping frequency: A few times a month vs More than once a week | 1.01 (0.93,1.1) |
| Shopping frequency: Once a month vs More than once a week | 0.87 (0.66,1.14) |
| Shopping frequency: Never vs More than once a week | 0.44** (0.26,0.73) |
| Shopping responsibility: Most of it/all of it vs None of it/some of it | 0.93* (0.88,0.99) |
| Number of people in household: 2 vs 1 | 1.47** (1.39,1.55) |
| Number of people in household: 3 vs 1 | 1.84** (1.74,1.96) |
| Number of people in household: More than 3 vs 1 | 2.39** (2.25,2.54) |
| Time of day: Lunch vs Morning | 0.99 (0.94,1.04) |
| Time of day: Dinner vs Morning | 0.97 (0.93,1.02) |
| Time of day: Night vs Morning | 1.05 (0.99,1.12) |
| Day of the week: Tuesday vs Monday | 1.02 (0.95,1.09) |
| Day of the week: Wednesday vs Monday | 1.05 (0.97,1.13) |
| Day of the week: Thursday vs Monday | 1.05 (0.98,1.11) |
| Day of the week: Friday vs Monday | 1.09* (1,1.18) |
| Day of the week: Saturday vs Monday | 1.07 (0.97,1.18) |
| Day of the week: Sunday vs Monday | 1.1* (1.02,1.19) |
| Deprivation Decile: 2 vs 1 | 1.01 (0.91,1.12) |
| Deprivation Decile: 3 vs 1 | 1.03 (0.95,1.11) |
| Deprivation Decile: 4 vs 1 | 0.99 (0.93,1.06) |
| Deprivation Decile: 5 vs 1 | 1.01 (0.95,1.08) |
| Deprivation Decile: 6 vs 1 | 0.99 (0.93,1.05) |
| Deprivation Decile: 7 vs 1 | 0.98 (0.92,1.05) |
| Deprivation Decile: 8 vs 1 | 0.99 (0.93,1.05) |
| Deprivation Decile: 9 vs 1 | 0.99 (0.94,1.05) |
| Deprivation Decile: 10 vs 1 | 0.98 (0.92,1.03) |
| Education: High school completed vs Less than high school | 1.08 (0.97,1.2) |
| Education: University degree vs Less than high school | 1.1+ (0.99,1.22) |
| Education: None of the above vs Less than high school | 1.16** (1.05,1.28) |
| BMI: Healthy weight vs Underweight | 1.13** (1.03,1.24) |
| BMI: Overweight vs Underweight | 1.19** (1.08,1.3) |
| BMI: Obese vs Underweight | 1.24** (1.13,1.36) |

Table S5. Full GLM model output for secondary outcome: total price of basket (£)

| **Primary Outcome: Total energy selected** | **Multiple Imputation Estimates (95%CI)** |
| --- | --- |
| (Intercept) | 7.17** (6.3,8.16) |
| NC vs UP | 1 (0.96,1.03) |
| NP vs UP | 1.01 (0.97,1.05) |
| NP vs NC | 1.01 (0.98,1.05) |
| Gender: Women vs Men | 1.08** (1.05,1.11) |
| Gender: Other vs Men | 0.9 (0.73,1.12) |
| Age: 25-34 vs 18-24 | 1.01 (0.95,1.06) |
| Age: 35-44 vs 18-34 | 1.01 (0.96,1.07) |
| Age: 45-54 vs 18-34 | 1.03 (0.98,1.09) |
| Age: 55-64 vs 18-34 | 1.14** (1.07,1.21) |
| Age: 65 and over vs 18-34 | 1.09* (1.02,1.17) |
| Income: £20,000- £39,999 vs under £20,000 | 1.04* (1,1.08) |
| Income: £40,000 - £59,999 vs under £20,000 | 1.05* (1,1.1) |
| Income: Over £60,000 vs under £20,000 | 1.11** (1.06,1.16) |
| Location: North vs London | 1 (0.95,1.06) |
| Location: South and East vs London | 1.01 (0.96,1.06) |
| Location: Midland vs London | 1 (0.95,1.06) |
| Location: Wales, Scotland and Northern Ireland vs London | 0.95 (0.88,1.04) |
| Urban: Rural vs Urban | 1.05* (1.01,1.1) |
| Urban: Suburban vs Urban | 1.04* (1.01,1.08) |
| Ethnicity: White Other vs White British | 1.11** (1.03,1.19) |
| Ethnicity: Asian or Asian British vs White British | 0.96 (0.91,1.03) |
| Ethnicity: Black vs White British | 1.05 (0.98,1.13) |
| Ethnicity: Mixed vs White British | 1.13* (1.02,1.24) |
| Ethnicity: Other vs White British | 1.01 (0.85,1.2) |
| Shopping frequency: Once a week vs More than once a week | 0.91** (0.86,0.96) |
| Shopping frequency: A few times a month vs More than once a week | 0.99 (0.93,1.06) |
| Shopping frequency: Once a month vs More than once a week | 0.76** (0.62,0.93) |
| Shopping frequency: Never vs More than once a week | 0.8 (0.5,1.28) |
| Shopping responsibility: Most of it/all of it vs None of it/some of it | 0.95* (0.91,0.99) |
| Number of people in household: 2 vs 1 | 1.48** (1.41,1.55) |
| Number of people in household: 3 vs 1 | 1.79** (1.71,1.89) |
| Number of people in household: More than 3 vs 1 | 2.2** (2.1,2.31) |
| Time of day: Lunch vs Morning | 1.01 (0.97,1.05) |
| Time of day: Dinner vs Morning | 1.02 (0.98,1.06) |
| Time of day: Night vs Morning | 1.07** (1.02,1.12) |
| Day of the week: Tuesday vs Monday | 1.02 (0.97,1.07) |
| Day of the week: Wednesday vs Monday | 1.03 (0.97,1.09) |
| Day of the week: Thursday vs Monday | 1.02 (0.97,1.07) |
| Day of the week: Friday vs Monday | 1.06+ (1,1.13) |
| Day of the week: Saturday vs Monday | 1.07 (0.99,1.15) |
| Day of the week: Sunday vs Monday | 1.05 (0.99,1.12) |
| Deprivation Decile: 2 vs 1 | 1 (0.92,1.09) |
| Deprivation Decile: 3 vs 1 | 1.05 (0.98,1.12) |
| Deprivation Decile: 4 vs 1 | 1.01 (0.95,1.06) |
| Deprivation Decile: 5 vs 1 | 1.04 (0.99,1.1) |
| Deprivation Decile: 6 vs 1 | 0.99 (0.94,1.04) |
| Deprivation Decile: 7 vs 1 | 1 (0.95,1.05) |
| Deprivation Decile: 8 vs 1 | 0.98 (0.94,1.03) |
| Deprivation Decile: 9 vs 1 | 0.98 (0.94,1.03) |
| Deprivation Decile: 10 vs 1 | 0.99 (0.95,1.04) |
| Education: High school completed vs Less than high school | 1.06 (0.98,1.16) |
| Education: University degree vs Less than high school | 1.08+ (0.99,1.18) |
| Education: None of the above vs Less than high school | 1.16** (1.07,1.27) |
| BMI: Healthy weight vs Underweight | 1.16** (1.09,1.24) |
| BMI: Overweight vs Underweight | 1.2** (1.12,1.28) |
| BMI: Obese vs Underweight | 1.21** (1.13,1.29) |

Table S6. Full GLM model output for secondary outcome: policy support

| **Primary Outcome: Total energy selected** | **Multiple Imputation Estimates (95%CI)** |
| --- | --- |
| (Intercept) | 2.17** (1.96,2.37) |
| NC vs UP | -0.28** (-0.34,-0.23) |
| NP vs UP | -0.51** (-0.56,-0.45) |
| NP vs NC | -0.22** (-0.28,-0.17) |
| Gender: Women vs Men | 0.01 (-0.04,0.06) |
| Gender: Other vs Men | 0.14 (-0.21,0.5) |
| Age: 25-34 vs 18-24 | 0.16** (0.08,0.25) |
| Age: 35-44 vs 18-34 | 0.22** (0.13,0.31) |
| Age: 45-54 vs 18-34 | 0.19** (0.1,0.28) |
| Age: 55-64 vs 18-34 | 0.16** (0.06,0.26) |
| Age: 65 and over vs 18-34 | 0.14* (0.03,0.25) |
| Income: £20,000- £39,999 vs under £20,000 | 0.04 (-0.02,0.11) |
| Income: £40,000 - £59,999 vs under £20,000 | 0.09* (0.02,0.16) |
| Income: Over £60,000 vs under £20,000 | 0.1* (0.02,0.18) |
| Location: North vs London | 0.04 (-0.04,0.13) |
| Location: South and East vs London | 0.06 (-0.02,0.15) |
| Location: Midland vs London | 0.05 (-0.04,0.14) |
| Location: Wales, Scotland and Northern Ireland vs London | 0.1 (-0.04,0.23) |
| Urban: Rural vs Urban | -0.04 (-0.12,0.03) |
| Urban: Suburban vs Urban | 0 (-0.06,0.06) |
| Ethnicity: White Other vs White British | -0.1+ (-0.22,0.01) |
| Ethnicity: Asian or Asian British vs White British | -0.06 (-0.16,0.04) |
| Ethnicity: Black vs White British | 0.12* (0.01,0.24) |
| Ethnicity: Mixed vs White British | 0.14+ (-0.02,0.3) |
| Ethnicity: Other vs White British | -0.13 (-0.41,0.14) |
| Shopping frequency: Once a week vs More than once a week | 0.01 (-0.07,0.1) |
| Shopping frequency: A few times a month vs More than once a week | 0.03 (-0.08,0.13) |
| Shopping frequency: Once a month vs More than once a week | 0.06 (-0.27,0.39) |
| Shopping frequency: Never vs More than once a week | 0.3 (-0.46,1.07) |
| Shopping responsibility: Most of it/all of it vs None of it/some of it | 0.09* (0.01,0.16) |
| Number of people in household: 2 vs 1 | 0.05 (-0.03,0.12) |
| Number of people in household: 3 vs 1 | 0.06 (-0.02,0.14) |
| Number of people in household: More than 3 vs 1 | 0.04 (-0.04,0.12) |
| Time of day: Lunch vs Morning | -0.03 (-0.09,0.03) |
| Time of day: Dinner vs Morning | -0.04 (-0.1,0.02) |
| Time of day: Night vs Morning | 0 (-0.08,0.08) |
| Day of the week: Tuesday vs Monday | 0.03 (-0.05,0.12) |
| Day of the week: Wednesday vs Monday | 0 (-0.1,0.1) |
| Day of the week: Thursday vs Monday | 0.02 (-0.06,0.1) |
| Day of the week: Friday vs Monday | -0.03 (-0.13,0.07) |
| Day of the week: Saturday vs Monday | 0.07 (-0.06,0.2) |
| Day of the week: Sunday vs Monday | 0.03 (-0.07,0.13) |
| Deprivation Decile: 2 vs 1 | 0.02 (-0.11,0.16) |
| Deprivation Decile: 3 vs 1 | -0.14* (-0.24,-0.03) |
| Deprivation Decile: 4 vs 1 | 0 (-0.08,0.09) |
| Deprivation Decile: 5 vs 1 | -0.03 (-0.11,0.05) |
| Deprivation Decile: 6 vs 1 | 0.03 (-0.05,0.11) |
| Deprivation Decile: 7 vs 1 | 0 (-0.08,0.08) |
| Deprivation Decile: 8 vs 1 | 0.01 (-0.07,0.09) |
| Deprivation Decile: 9 vs 1 | -0.01 (-0.08,0.06) |
| Deprivation Decile: 10 vs 1 | -0.01 (-0.07,0.06) |
| Education: High school completed vs Less than high school | -0.01 (-0.14,0.12) |
| Education: University degree vs Less than high school | 0.02 (-0.1,0.15) |
| Education: None of the above vs Less than high school | 1.16** (1.07,1.27) |
| BMI: Healthy weight vs Underweight | 1.16** (1.09,1.24) |
| BMI: Overweight vs Underweight | 1.2** (1.12,1.28) |
| BMI: Obese vs Underweight | 1.21** (1.13,1.29) |

Table S7. Full GLM model output for exploratory outcome: number of items in basket

| **Primary Outcome: Total energy selected** | **Multiple Imputation Estimates (95%CI)** |
| --- | --- |
| (Intercept) | 4.65** (4.33,4.98) |
| NC vs UP | 0.99 (0.97,1.01) |
| NP vs UP | 0.98** (0.96,0.99) |
| NP vs NC | 0.99+ (0.97,1) |
| Gender: Women vs Men | 1.22** (1.2,1.24) |
| Gender: Other vs Men | 1.11* (1.01,1.22) |
| Age: 25-34 vs 18-24 | 1.03* (1,1.05) |
| Age: 35-44 vs 18-34 | 1.06** (1.04,1.09) |
| Age: 45-54 vs 18-34 | 1.11** (1.08,1.14) |
| Age: 55-64 vs 18-34 | 1.27** (1.23,1.3) |
| Age: 65 and over vs 18-34 | 1.18** (1.14,1.22) |
| Income: £20,000- £39,999 vs under £20,000 | 1.01 (1,1.03) |
| Income: £40,000 - £59,999 vs under £20,000 | 1.03* (1.01,1.05) |
| Income: Over £60,000 vs under £20,000 | 1.06** (1.04,1.08) |
| Location: North vs London | 1.03* (1,1.05) |
| Location: South and East vs London | 1.03** (1.01,1.06) |
| Location: Midland vs London | 1.06** (1.03,1.08) |
| Location: Wales, Scotland and Northern Ireland vs London | 1 (0.96,1.04) |
| Urban: Rural vs Urban | 1.1** (1.08,1.12) |
| Urban: Suburban vs Urban | 1.06** (1.04,1.07) |
| Ethnicity: White Other vs White British | 1.11** (1.08,1.14) |
| Ethnicity: Asian or Asian British vs White British | 0.95** (0.92,0.98) |
| Ethnicity: Black vs White British | 1.09** (1.06,1.12) |
| Ethnicity: Mixed vs White British | 1.03 (0.99,1.08) |
| Ethnicity: Other vs White British | 1 (0.92,1.08) |
| Shopping frequency: Once a week vs More than once a week | 0.95** (0.93,0.97) |
| Shopping frequency: A few times a month vs More than once a week | 0.96** (0.93,0.99) |
| Shopping frequency: Once a month vs More than once a week | 0.81** (0.73,0.9) |
| Shopping frequency: Never vs More than once a week | 0.8+ (0.62,1.03) |
| Shopping responsibility: Most of it/all of it vs None of it/some of it | 0.93** (0.91,0.94) |
| Number of people in household: 2 vs 1 | 1.37** (1.34,1.4) |
| Number of people in household: 3 vs 1 | 1.66** (1.61,1.7) |
| Number of people in household: More than 3 vs 1 | 2.01** (1.96,2.06) |
| Time of day: Lunch vs Morning | 0.97** (0.96,0.99) |
| Time of day: Dinner vs Morning | 0.99+ (0.97,1) |
| Time of day: Night vs Morning | 1.07** (1.05,1.1) |
| Day of the week: Tuesday vs Monday | 0.99 (0.97,1.01) |
| Day of the week: Wednesday vs Monday | 1.01 (0.98,1.04) |
| Day of the week: Thursday vs Monday | 1.02 (1,1.04) |
| Day of the week: Friday vs Monday | 1.04** (1.01,1.07) |
| Day of the week: Saturday vs Monday | 1.04* (1.01,1.08) |
| Day of the week: Sunday vs Monday | 1.09** (1.06,1.12) |
| Deprivation Decile: 2 vs 1 | 1.02 (0.98,1.06) |
| Deprivation Decile: 3 vs 1 | 1.07** (1.04,1.11) |
| Deprivation Decile: 4 vs 1 | 1 (0.97,1.02) |
| Deprivation Decile: 5 vs 1 | 1.07** (1.05,1.1) |
| Deprivation Decile: 6 vs 1 | 0.98 (0.96,1.01) |
| Deprivation Decile: 7 vs 1 | 0.96** (0.94,0.99) |
| Deprivation Decile: 8 vs 1 | 1 (0.98,1.02) |
| Deprivation Decile: 9 vs 1 | 0.97** (0.95,0.99) |
| Deprivation Decile: 10 vs 1 | 0.98* (0.96,1) |
| Education: High school completed vs Less than high school | 1.02 (0.96,1.07) |
| Education: University degree vs Less than high school | 1.05+ (0.99,1.11) |
| Education: None of the above vs Less than high school | 1.11** (1.05,1.17) |
| BMI: Healthy weight vs Underweight | 1.14** (1.1,1.17) |
| BMI: Overweight vs Underweight | 1.17** (1.13,1.21) |
| BMI: Obese vs Underweight | 1.2** (1.16,1.24) |

Table S8. Full GLM model output for exploratory outcome: proportion of basket that is HFSS items

| **Primary Outcome: Total energy selected** | **Multiple Imputation Estimates (95%CI)** |
| --- | --- |
| (Intercept) | 0.38** (0.33,0.43) |
| NC vs UP | -0.01 (-0.02,0) |
| NP vs UP | 0 (-0.02,0.01) |
| NP vs NC | 0.01 (-0.01,0.02) |
| Gender: Women vs Men | -0.03** (-0.04,-0.02) |
| Gender: Other vs Men | 0.06 (-0.02,0.15) |
| Age: 25-34 vs 18-24 | -0.04** (-0.06,-0.02) |
| Age: 35-44 vs 18-34 | -0.06** (-0.08,-0.04) |
| Age: 45-54 vs 18-34 | -0.09** (-0.11,-0.07) |
| Age: 55-64 vs 18-34 | -0.11** (-0.13,-0.08) |
| Age: 65 and over vs 18-34 | -0.14** (-0.16,-0.11) |
| Income: £20,000- £39,999 vs under £20,000 | -0.03** (-0.04,-0.01) |
| Income: £40,000 - £59,999 vs under £20,000 | -0.03** (-0.05,-0.01) |
| Income: Over £60,000 vs under £20,000 | -0.04** (-0.06,-0.02) |
| Location: North vs London | -0.01 (-0.04,0.01) |
| Location: South and East vs London | 0 (-0.02,0.02) |
| Location: Midland vs London | -0.01 (-0.03,0.01) |
| Location: Wales, Scotland and Northern Ireland vs London | -0.02 (-0.05,0.01) |
| Urban: Rural vs Urban | -0.02* (-0.04,0) |
| Urban: Suburban vs Urban | -0.01+ (-0.03,0) |
| Ethnicity: White Other vs White British | -0.03* (-0.06,0) |
| Ethnicity: Asian or Asian British vs White British | -0.02 (-0.04,0.01) |
| Ethnicity: Black vs White British | -0.01 (-0.04,0.02) |
| Ethnicity: Mixed vs White British | -0.02 (-0.06,0.02) |
| Ethnicity: Other vs White British | -0.02 (-0.09,0.05) |
| Shopping frequency: Once a week vs More than once a week | -0.01 (-0.03,0.01) |
| Shopping frequency: A few times a month vs More than once a week | 0 (-0.03,0.02) |
| Shopping frequency: Once a month vs More than once a week | -0.02 (-0.1,0.06) |
| Shopping frequency: Never vs More than once a week | -0.14 (-0.32,0.05) |
| Shopping responsibility: Most of it/all of it vs None of it/some of it | 0.01 (-0.01,0.03) |
| Number of people in household: 2 vs 1 | 0.02+ (0,0.03) |
| Number of people in household: 3 vs 1 | 0.03* (0.01,0.05) |
| Number of people in household: More than 3 vs 1 | 0.05** (0.03,0.07) |
| Time of day: Lunch vs Morning | 0.01+ (0,0.03) |
| Time of day: Dinner vs Morning | 0.02* (0,0.03) |
| Time of day: Night vs Morning | 0.01 (0,0.03) |
| Day of the week: Tuesday vs Monday | -0.02+ (-0.04,0) |
| Day of the week: Wednesday vs Monday | -0.02 (-0.04,0.01) |
| Day of the week: Thursday vs Monday | -0.01 (-0.03,0.01) |
| Day of the week: Friday vs Monday | -0.02+ (-0.05,0) |
| Day of the week: Saturday vs Monday | 0 (-0.03,0.03) |
| Day of the week: Sunday vs Monday | -0.01 (-0.04,0.01) |
| Deprivation Decile: 2 vs 1 | -0.02 (-0.06,0.01) |
| Deprivation Decile: 3 vs 1 | 0 (-0.02,0.03) |
| Deprivation Decile: 4 vs 1 | -0.01 (-0.03,0.01) |
| Deprivation Decile: 5 vs 1 | -0.02+ (-0.04,0) |
| Deprivation Decile: 6 vs 1 | -0.01 (-0.03,0.01) |
| Deprivation Decile: 7 vs 1 | 0.01 (-0.01,0.03) |
| Deprivation Decile: 8 vs 1 | 0 (-0.02,0.02) |
| Deprivation Decile: 9 vs 1 | 0 (-0.02,0.02) |
| Deprivation Decile: 10 vs 1 | 0.01 (-0.01,0.02) |
| Education: High school completed vs Less than high school | -0.02 (-0.05,0.02) |
| Education: University degree vs Less than high school | -0.01 (-0.04,0.02) |
| Education: None of the above vs Less than high school | -0.03+ (-0.06,0) |
| BMI: Healthy weight vs Underweight | -0.03** (-0.06,-0.01) |
| BMI: Overweight vs Underweight | -0.03* (-0.06,-0.01) |
| BMI: Obese vs Underweight | -0.02+ (-0.05,0) |

Table S9. Full GLM model output for exploratory outcome: calorie density of basket

| **Primary Outcome: Total energy selected** | **Multiple Imputation Estimates (95%CI)** |
| --- | --- |
| (Intercept) | 2.07** (1.2,2.94) |
| NC vs UP | -0.05 (-0.3,0.2) |
| NP vs UP | 0.12 (-0.13,0.36) |
| NP vs NC | 0.17 (-0.08,0.41) |
| Gender: Women vs Men | -0.33** (-0.54,-0.11) |
| Gender: Other vs Men | -0.11 (-1.64,1.42) |
| Age: 25-34 vs 18-24 | -0.02 (-0.39,0.34) |
| Age: 35-44 vs 18-34 | 0.09 (-0.28,0.46) |
| Age: 45-54 vs 18-34 | -0.13 (-0.51,0.26) |
| Age: 55-64 vs 18-34 | 0.05 (-0.37,0.47) |
| Age: 65 and over vs 18-34 | -0.04 (-0.51,0.43) |
| Income: £20,000- £39,999 vs under £20,000 | -0.02 (-0.29,0.26) |
| Income: £40,000 - £59,999 vs under £20,000 | -0.03 (-0.34,0.28) |
| Income: Over £60,000 vs under £20,000 | 0.2 (-0.14,0.53) |
| Location: North vs London | -0.02 (-0.39,0.35) |
| Location: South and East vs London | 0.02 (-0.34,0.38) |
| Location: Midland vs London | 0.23 (-0.16,0.62) |
| Location: Wales, Scotland and Northern Ireland vs London | -0.02 (-0.6,0.55) |
| Urban: Rural vs Urban | -0.24 (-0.55,0.07) |
| Urban: Suburban vs Urban | -0.29* (-0.54,-0.04) |
| Ethnicity: White Other vs White British | 0.02 (-0.48,0.52) |
| Ethnicity: Asian or Asian British vs White British | 0.62** (0.18,1.06) |
| Ethnicity: Black vs White British | 0.12 (-0.37,0.61) |
| Ethnicity: Mixed vs White British | -0.07 (-0.77,0.62) |
| Ethnicity: Other vs White British | -0.16 (-1.35,1.04) |
| Shopping frequency: Once a week vs More than once a week | -0.1 (-0.47,0.26) |
| Shopping frequency: A few times a month vs More than once a week | -0.08 (-0.53,0.38) |
| Shopping frequency: Once a month vs More than once a week | 0.04 (-1.38,1.45) |
| Shopping frequency: Never vs More than once a week | -1.44 (-4.71,1.84) |
| Shopping responsibility: Most of it/all of it vs None of it/some of it | -0.02 (-0.33,0.29) |
| Number of people in household: 2 vs 1 | 0.16 (-0.16,0.49) |
| Number of people in household: 3 vs 1 | 0.23 (-0.12,0.59) |
| Number of people in household: More than 3 vs 1 | 0.59** (0.25,0.93) |
| Time of day: Lunch vs Morning | 0.15 (-0.12,0.42) |
| Time of day: Dinner vs Morning | -0.05 (-0.32,0.22) |
| Time of day: Night vs Morning | -0.01 (-0.36,0.33) |
| Day of the week: Tuesday vs Monday | 0.03 (-0.34,0.41) |
| Day of the week: Wednesday vs Monday | 0.07 (-0.34,0.49) |
| Day of the week: Thursday vs Monday | 0.18 (-0.17,0.52) |
| Day of the week: Friday vs Monday | 0.07 (-0.37,0.5) |
| Day of the week: Saturday vs Monday | 0.08 (-0.47,0.64) |
| Day of the week: Sunday vs Monday | 0 (-0.43,0.43) |
| Deprivation Decile: 2 vs 1 | -0.05 (-0.62,0.52) |
| Deprivation Decile: 3 vs 1 | 0 (-0.45,0.45) |
| Deprivation Decile: 4 vs 1 | 0.04 (-0.33,0.42) |
| Deprivation Decile: 5 vs 1 | -0.1 (-0.46,0.26) |
| Deprivation Decile: 6 vs 1 | -0.13 (-0.46,0.21) |
| Deprivation Decile: 7 vs 1 | 0.05 (-0.29,0.39) |
| Deprivation Decile: 8 vs 1 | 0.1 (-0.23,0.44) |
| Deprivation Decile: 9 vs 1 | -0.11 (-0.42,0.19) |
| Deprivation Decile: 10 vs 1 | 0.08 (-0.2,0.37) |
| Education: High school completed vs Less than high school | 0.01 (-0.54,0.55) |
| Education: University degree vs Less than high school | -0.08 (-0.61,0.45) |
| Education: None of the above vs Less than high school | 0.01 (-0.54,0.56) |
| BMI: Healthy weight vs Underweight | -0.13 (-0.57,0.31) |
| BMI: Overweight vs Underweight | -0.15 (-0.6,0.31) |
| BMI: Obese vs Underweight | 0.12 (-0.35,0.58) |

Table S10. Full GLM model with interaction terms output for exploratory outcome: moderating effects of gender on the effects of intervention on total energy (kcal) in basket

| **Primary Outcome: Total energy selected** | **Multiple Imputation Estimates (95%CI)** |
| --- | --- |
| (Intercept) | 8.34*** (8.16, 8.52) |
| NC vs UP | -0.02 (-0.08, 0.04) |
| NP vs UP | -0.06* (-0.12, 0) |
| NP vs NC | -0.04 (-0.1, 0.02) |
| Gender: Men vs Women | -0.22*** (-0.28, -0.16) |
| Age: 25-34 vs 18-24 | 0.01 (-0.07, 0.09) |
| Age: 35-44 vs 18-34 | 0.06 (-0.02, 0.14) |
| Age: 45-54 vs 18-34 | 0.08* (0.00, 0.16) |
| Age: 55-64 vs 18-34 | 0.24*** (0.16, 0.32) |
| Age: 65 and over vs 18-34 | 0.19*** (0.1, 0.27) |
| Income: £20,000- £39,999 vs under £20,000 | 0.01 (-0.05, 0.07) |
| Income: £40,000 - £59,999 vs under £20,000 | -0.01 (-0.07, 0.05) |
| Income: Over £60,000 vs under £20,000 | 0.01 (-0.05, 0.07) |
| Location: North vs London | 0.06 (-0.02, 0.14) |
| Location: South and East vs London | 0.08* (0, 0.16) |
| Location: Midland vs London | 0.07 (-0.01, 0.15) |
| Location: Wales, Scotland and Northern Ireland vs London | 0.04 (-0.08, 0.16) |
| Urban: Rural vs Urban | 0.08* (0.02, 0.14) |
| Urban: Suburban vs Urban | 0.06* (0, 0.12) |
| Ethnicity: White Other vs White British | 0.02 (-0.06, 0.1) |
| Ethnicity: Asian or Asian British vs White British | -0.01 (-0.13, 0.11) |
| Ethnicity: Black vs White British | 0.14* (0.04, 0.24) |
| Ethnicity: Mixed vs White British | 0.1 (-0.04, 0.24) |
| Ethnicity: Other vs White British | -0.12 (-0.34, 0.1) |
| Shopping frequency: Once a week vs More than once a week | 0.00 (-0.06, 0.06) |
| Shopping frequency: A few times a month vs More than once a week | 0.01 (-0.07, 0.09) |
| Shopping frequency: Once a month vs More than once a week | -0.14 (-0.41, 0.13) |
| Shopping frequency: Never vs More than once a week | -0.86*** (-1.37, -0.35) |
| Shopping responsibility: Most of it/all of it vs None of it/some of it | -0.07* (-0.13, -0.01) |
| Number of people in household: 2 vs 1 | 0.39*** (0.33, 0.45) |
| Number of people in household: 3 vs 1 | 0.62*** (0.56, 0.68) |
| Number of people in household: More than 3 vs 1 | 0.87*** (0.81, 0.93) |
| Time of day: Lunch vs Morning | -0.01 (-0.07, 0.05) |
| Time of day: Dinner vs Morning | -0.03 (-0.07, 0.01) |
| Time of day: Night vs Morning | 0.05 (-0.01, 0.11) |
| Day of the week: Tuesday vs Monday | 0.02 (-0.04, 0.08) |
| Day of the week: Wednesday vs Monday | 0.05 (-0.03, 0.13) |
| Day of the week: Thursday vs Monday | 0.05 (-0.01, 0.11) |
| Day of the week: Friday vs Monday | 0.09* (0.01, 0.17) |
| Day of the week: Saturday vs Monday | 0.06 (-0.04, 0.16) |
| Day of the week: Sunday vs Monday | 0.10* (0.02, 0.18) |
| Deprivation Decile: 2 vs 1 | 0.01 (-0.09, 0.11) |
| Deprivation Decile: 3 vs 1 | 0.03 (-0.05, 0.11) |
| Deprivation Decile: 4 vs 1 | -0.01 (-0.07, 0.05) |
| Deprivation Decile: 5 vs 1 | 0.01 (-0.05, 0.07) |
| Deprivation Decile: 6 vs 1 | -0.02 (-0.08, 0.04) |
| Deprivation Decile: 7 vs 1 | -0.02 (-0.08, 0.04) |
| Deprivation Decile: 8 vs 1 | -0.02 (-0.08, 0.04) |
| Deprivation Decile: 9 vs 1 | 0.00 (-0.06, 0.06) |
| Deprivation Decile: 10 vs 1 | -0.03 (-0.09, 0.03) |
| Education: High school completed vs Less than high school | 0.08 (-0.02, 0.18) |
| Education: University degree vs Less than high school | 0.10 (0.00, 0.20) |
| Education: None of the above vs Less than high school | 0.14* (0.02, 0.26) |
| BMI: Healthy weight vs Underweight | 0.12* (0.02, 0.22) |
| BMI: Overweight vs Underweight | 0.17*** (0.07, 0.27) |
| BMI: Obese vs Underweight | 0.21*** (0.11, 0.31) |
| NC*Men vs UP*Women | 0.00 (-0.1, 0.10) |
| NP*Men vs UP*Women | 0.10 (0.00, 0.20) |
